# Strengthening health systems for universal health coverage and sustainable development

**DOI:** 10.2471/BLT.16.187476

**Published:** 2017-04-07

**Authors:** Marie Paule Kieny, Henk Bekedam, Delanyo Dovlo, James Fitzgerald, Jarno Habicht, Graham Harrison, Hans Kluge, Vivian Lin, Natela Menabde, Zafar Mirza, Sameen Siddiqi, Phyllida Travis

**Affiliations:** a Health Systems and Innovation, World Health Organization, avenue Appia 20, 1211 Geneva 27, Switzerland.; b India Country Office, World Health Organization, New Delhi, India.; c Regional Office for Africa, World Health Organization, Brazzaville, Congo.; d Pan American Health Organization, Washington, United States of America (USA).; e Kyrgyzstan Country Office, World Health Organization, Bishkek, Kyrgyzstan.; f Malaysia Country Office, World Health Organization, Kuala Lumpur, Malaysia.; g Regional Office for Europe, World Health Organization, Copenhagen, Denmark.; h Regional Office for Western Pacific, World Health Organization, Manila, Philippines.; i World Health Organization Office at the United Nations, World Health Organization, New York, USA.; j Islamic Republic of Iran Country Office, World Health Organization, Tehran, Islamic Republic of Iran.; k Regional Office for South-East Asia, World Health Organization, New Delhi, India.

The *2030 agenda for sustainable development* is an opportunity for governments and the international community to renew their commitment to improving health as a central component of development.^1^ The accompanying 17 sustainable development goals (SDGs) define the priority areas of action.^2^ Goal 3 (to ensure healthy lives and promote well-being for all at all ages), with Target 3.8 on universal health coverage (UHC), emphasize the importance of all people and communities having access to quality health services without risking financial hardship.^2^ These health services include those targeting individuals, such as curative care and population-based services, such as health promotion.^3^

Achieving UHC is an important objective for all countries to attain equitable and sustainable health outcomes and improve the well-being of individuals and communities.^4^^,^^5^ Health system strengthening is a means to progress towards UHC. A functioning health system is organized around the people, institutions and resources that are mandated to improve, maintain or restore the health of a given population. Health system strengthening refers to significant and purposeful effort to improve the system’s performance.^6^ Strengthening is one way to ensure that the system’s performance embodies the intermediary objectives of most national health policies, plans and strategies – quality, equity, efficiency, accountability, resilience and sustainability (Box 1).

Box 1 **Intermediary objectives of national health policies, plans and strategies**

**Quality**

Health-care quality is the extent to which health services provided to individuals and patient populations improve desired health outcomes, consistent with current professional knowledge.^7^

**Equity**

Equity in health is a measure of the degree to which health policies can fairly distribute well-being in the population. It can also refer to the absence of systematic or remediable differences in health status or access to health care.^8^

**Efficiency**

Efficiency refers to the capacity to produce maximum output for a given input.^8^

**Accountability**

Accountability results from processes in the health system that ensure health-care actors^a^ to take responsibility for what they are obliged to do and are answerable for their actions.^8^

**Resilience**

Health system resilience is the capacity of health-care actors,^a^ institutions and populations to prepare for and respond to crises, maintain core functions in time of crisis; and, informed by lessons learnt during the crisis, reorganize if needed.^9^

**Sustainability**

Sustainability refers to the potential for maintaining beneficial outcomes for an agreed period of time at an acceptable level of resource commitment.^8^

^a^ Health-care actors are individuals or groups with an interest in the health system, including patients and their families, nurses, physicians, laboratorial technical staff, and other external entities such as regulators, insurance companies and health-care organizations.

**Fig. 1:**
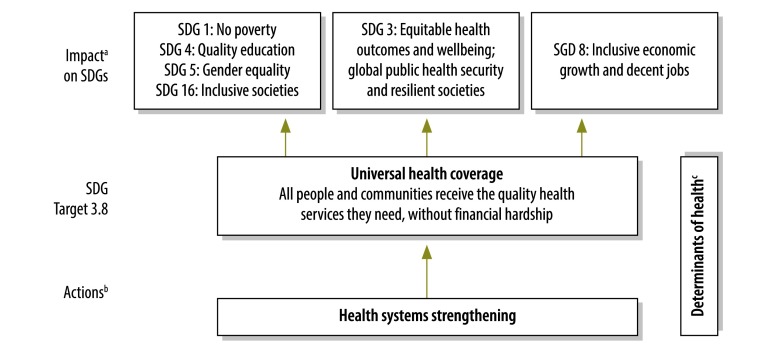
How health system strengthening contributes to sustainable development goals through universal health coverage

We argue that UHC contributes to the SDGs in several ways. The impact of health system strengthening on UHC, and how health system strengthening, through UHC, contributes to different sustainable development goals is illustrated in Fig. 1.

SDG: sustainable development goal.

^a ^A health impact can be positive or negative. A positive impact is an effect which contributes to good health or improvement in health status. A negative impact causes or contributes to ill health.^8^

^b^ Action refers to interventions that aim at strengthening a health system. ^c ^Whether people are healthy or not, is determined by their circumstances and environment. The determinants of health include the social and economic environment, the physical environment, and the person’s individual characteristics and behaviours.^10^

One way UHC contributes to the SDGs is by promoting global public health security and it does so by increasing the resilience of health systems to respond to health threats that spread within as well as across national borders.^6^^,^^11^ The 2012 Middle East Respiratory Syndrome coronavirus, the 2013–2016 Ebola virus disease and 2015 Zika virus outbreaks prompted the international community of the financial aftermath many countries faced as a result of protracted health emergencies. The impact of humanitarian and natural disasters is exacerbated by weak health systems.^12^ These recent outbreaks showed that resilience is an important feature of a health system and its effect on health workers’ ability to adapt and effectively address complex challenges when responding to emergencies. Resilience should be envisaged as a critical objective of contemporary health system reforms.^13^ When compared to resources spent on emergency responses, it is cost-efficient and in the long-term sustainable to invest in building resilient and functioning health systems.

We claim that progress towards UHC will be essential to four specific SDG goals and the pledge to leave no one behind. First, as adults in poor health are more likely to be unemployed, when investments are made in improving health outcomes for the entire population, this can also contribute to SDG 1 (end poverty in all its forms everywhere). In addition, implementation of social protection systems to address out-of-pocket health expenditure reduces the incidence of catastrophic or impoverishing household health spending. Second, given that children and adolescents with good health have better educational outcomes, health has an important role to play in advancing SDG 4 (ensure inclusive and equitable education and promote lifelong learning opportunities for all). Third, as women comprise over 75% of the health workforce in many countries,^14^ the health system can contribute to advancing SDG 5 (achieve gender equality and empower all women and girls). Fourth, through the development of health systems that create fair, trustworthy and responsive social institutions, health system strengthening directly contributes to SDG 16 (promote inclusive societies for sustainable development, provide access to justice for all and build effective, accountable and inclusive institutions for all).

Investments in the health sector to support UHC will boost economic growth in line with SDG 8 (promote sustained, inclusive and sustainable economic growth, full and productive employment and decent work for all). The WHO report from the High-level Commission on Health Employment and Economic Growth states that the contribution to economic growth can happen through six inter-related pathways.^15^

The first pathway is through investment in health which contributes to an increase in life expectancy and healthier workers, contributing to increases in economic productivity. The Lancet Commission on Investing in Health reported that around one quarter of economic growth between 2000 and 2011 in low- and middle-income countries resulted from the value added by improvements in the health of the population. The estimated return on investment in health from improved economic growth was nine to one.^16^

The second pathway is through promoting economic output. The health sector adds direct economic value by expanding the number of jobs, investing in infrastructure projects and purchasing supplies needed for health-care delivery. A rapid and unprecedented growth in global health employment of around 40 million new jobs, mostly in middle- and high-income countries, is expected by 2030.^15^This growth will happen against a backdrop of 201 million unemployed people in 2014. By 2020, the number of unemployed may increase and because of technological advances from the fourth industrial revolution, it is expected that 7.1 million jobs will become redundant.^17^ Given that occupations in the health and social sector are more labour intensive and less likely to be automated, the health sector will be an even more important source of employment in the future.

Third is through enhancing social protection. Investing in decent jobs in the health sector contributes to enhancing social protection systems, for example in case of sickness, disability, unemployment and old age, as well as financial protection against loss of income, out-of-pocket payments and catastrophic health expenditures. Social protection in turn, promotes sustainable pro-poor economic growth.^18^

The fourth pathway is linked to social cohesion. Equal societies are more economically productive societies.^19^

Fifth is through promoting innovation and diversification. The production and export of pharmaceuticals, equipment and medical services has been an important driver of economic growth in many countries.^15^ Scientific and social innovations in this sector are likely to further support economic growth in the future.

The sixth pathway is by protecting and promoting human security. Strong health systems perform better in the detection, prevention and control of infectious disease outbreaks, protecting individual and global health security for peace, development, and economic growth.^6^ The expectation is that the health sector contribution to SDG 8, by protecting and promoting human security, will be significant.

To deliver its potential, effective UHC development will require financing and leadership. Even in fragile states and least developed countries, domestic resources contribute to about 75% of total health spending.^20^ However, these domestic resources are often not equitably distributed either geographically or among various income quintiles, with out-of-pocket expenditures remaining unacceptably high. In many countries, a narrow fiscal space will not allow a sharp increase in domestic funding, but it is possible to critically examine and then recast how and where funding is allocated and expenditures incurred.

To meet the health-related targets and make progress towards sustainable development, governments will need to use their domestic resources effectively, ensure people's interests are taken into consideration and that they have access to information and education. Governments also need to prioritize health prevention and promotion measures.

Numerous challenges currently exist for governments to overcome. In many countries, funding is disease-oriented with limited coordination among partners and alignment with national health strategies and plans is poor. Long-term sustainable investments in health systems have been neglected. Additionally, there are rigidities in the production and allocation of professional roles, and vested interests in the management of the health services. The SDGs provide an opportunity to overcome these challenges and build political commitment to a common health system strengthening agenda. Realizing progress towards UHC requires some level of guidance to promote a coherent and consolidated agenda for health system strengthening, which can be applied to country-specific UHC roadmaps.

While countries pursue their ongoing national efforts to strengthen their health systems, the same effort is being reinforced at regional and global level. In September 2016, the Director-General of the World Health Organization announced the establishment of a global platform, the International Health Partnership for UHC 2030, expanding the scope of IHP+ to include health system strengthening towards the achievement of UHC.^21^ IHP+ is a group of partners who work together to put international principles for development cooperation into practice in the health sector.^21^ The global platform aims to bring together development partners and governments, to improve coordination of health system strengthening efforts in countries, to facilitate multistakeholder policy dialogue, promote accountability and build political momentum around a shared and global vision of health system strengthening for UHC.
