# Corrigendum

**DOI:** 10.2471/BLT.17.100817

**Published:** 2017-08-01

In Volume 95, Issue 7, July 2017, page 537, the three last authors’ affiliations should be as follows: 

Zafar Mirza^j^, Sameen Siddiqi^k^ & Phyllida Travis^l^.

^j^ Regional Office for Eastern Mediterranean, World Health Organization, Cairo, Egypt.

^k^ Islamic Republic of Iran Country Office, World Health Organization, Tehran, Islamic Republic of Iran.

^l ^Regional Office for South-East Asia, World Health Organization, New Delhi, India.

